# Apoptosis induction and proliferation inhibition by silibinin encapsulated in nanoparticles in MIA PaCa-2 cancer cells and deregulation of some miRNAs

**DOI:** 10.22038/ijbms.2020.39427.9349

**Published:** 2020-04

**Authors:** Fatemeh Khakinezhad Tehrani, Najmeh Ranji, Fatemeh Kouhkan, Simzar Hosseinzadeh

**Affiliations:** 1 Department of Biology, Faculty of Sciences, Rasht Branch, Islamic Azad University, Rasht, Iran; 2 Stem cell Technology Research Center, Tehran, Iran; 3 Department of Tissue Engineering and Regenerative Medicine, School of Advanced Technologies in Medicine, Shahid Beheshti University of Medical Sciences, Tehran, Iran

**Keywords:** Cancer stem cell, miRNA, Nanoparticles, Pancreatic cancer, Silibinin

## Abstract

**Objective(s):**

Silibinin, as an herbal compound, has anti-cancer activity. Because of low solubility of silibinin in water and body fluids, it was encapsulated in polymersome nanoparticles and its effects were evaluated on pancreatic cancer cells and cancer stem cells.

**Materials and Methods:**

MIA PaCa-2 pancreatic cancer cells were treated with different doses of silibinin encapsulated in polymersome nanoparticles (SPNs). Stemness of MIA PaCa-2 cells was evaluated by hanging drop technique and CD133, CD24, and CD44 staining. The effects of SPNs on cell cycle, apoptosis and the expression of several genes and miRNAs were investigated.

**Results:**

IC_50_ of SPNs was determined to be 40 µg/ml after 24 hr. Our analysis showed that >98% of MIA PaCa-2 cells expressed three stem cell markers. FACS analysis showed a decrease in these markers in SPNs-treated cells. PI/AnnexinV staining revealed that 40 µg/ml and 50 µg/ml of SPNs increased apoptosis up to ~40% and >80% of treated cells, respectively. Upregulation of miR-34a, miR-126, and miR-let7b and downregulation of miR-155, miR-222 and miR-21 was observed in SPNs-treated cells. In addition, downregulation of some genes involved in proliferation or migration such as AKT3, MASPINE, and SERPINEA12, and upregulation of apoptotic genes were observed in treated cells.

**Conclusion:**

Our results suggested that SPNs induced apoptosis and inhibited migration and proliferation in pancreatic cells and cancer stem cells through suppression of some onco-miRs and induction of some tumor suppressive miRs, as well as their targets.

## Introduction

Pancreatic cancer is one of the most common reasons for cancer-related mortality worldwide (1, 2), while most patients die within one year after diagnosis and there is median survival in patients whose disease has been diagnosed in less than six months (3). Despite using different drugs to eliminate cancer cells, a small population in the cancer tissue with ability of self-renewal and pluripotency (4), called cancer stem cells (CSCs), are the cause of resistance to chemotherapy and radiotherapy. CSCs contribute to tumor initiation, progression and metastasis (5). Therefore, finding new strategies helps overcome the immortality of CSCs.

Silibinin is the major active component of silymarin (1), which is extracted from fruits and seeds of *Silybum marianum* (milk thistle) (6). Hepatoprotective, anti-inflammatory, antioxidant and anti-cancer effects of silibinin and silymarin were defined in various studies (6). The effects of silymarin against cirrhosis, jaundice and hepatitis have been proved. In addition, it has been determined that milk thistle enhances bile flow and removes liver and spleen obstructions (6). Different reports revealed that silibinin has effects on various cancers such as pancreatic, prostate, lung, skin, breast, colon, renal, hepatic, cervical, ovarian and gastric carcinoma through different mechanisms (7). However, poor absorption is a problem for the use of this medicine (6). Nowadays, different carriers such as liposomes (8) however its poor aqueous solubility and bioavailability have to be overcome. In the current study curcumin is encapsulated in krill lipids-based liposomes (marinosomes, dendrimers, micelles, and nanoemulsions (9) are used to release further amount of insoluble drugs into cells. Nanoencapsulation of therapeutic agents increases their efficacy, specificity and targeting ability (10). Nanocarriers (NCs) protect their payload from premature degradation in the biological environment with higher bioavailability and prolonged presence in blood and cellular uptake (11). Polymersome is a nano-sized artificial vesicle made from amphiphilic block copolymers that can be used to deliver different molecules such as plasmids, proteins and compounds with low molecular weight into cells (12). Polymersomes are more stable and storable nanoparticles in comparison with liposomes and unlike micelles, polymersomes can encapsulate hydrophilic and hydrophobic biomaterials (13).

Recent studies have shown that the expression pattern of miRNAs are a rich source of pathognomonic tumor information compared to messenger RNA expression proﬁles (14) known as microRNAs (miRNAs. Furthermore, the expression patterns of miRNAs are extraordinarily unique to each tumor type and to their tissue of origin (15) The miRNAs are a family of highly conserved, non-coding, 17–25 nucleotide long RNA products that regulate gene expression at the post-transcriptional level (16); it is reasonable to assume that miRNAs are also involved in human diseases such as cancers. Several groups of miRNAs have been identified to regulate the expression of tumor-associated genes (17) Abnormal expression of miRNAs is associated with tumor promotion and one may inhibit the tumor by minimizing cell proliferation, survival and differentiation (14known as microRNAs (miRNAs,18)comparatively little is known about the genetics of papillary thyroid carcinoma (PTC. Hence, restoring the expression of such miRNAs in tumor cells can possibly promote differentiation and inhibit malignant cells proliferation and/or induce apoptosis (16). Thus, up/downregulation of miRNAs in cancerous cells can be indicative of their role as onco-miRs or tumor suppressive miRs (19). Downregulation of let-7b, miR-126 (20) and miR-34 (21) as tumor suppressive miRs had been found in tumor tissue. MiR-34 plays role in the regulation of p53 expression through repression of Sirtuin 1 (SIRT1), histone deacetylase 1 (HDAC1) and the transcriptional factor YY1 (21). In addition, overexpression of miR-34a induces cell cycle arrest and senescence, and inhibits cell growth (22)predicting disease outcome remains a major clinical challenge. Recent expression profiling studies in prostate cancer suggest microRNAs (miRNAs. Overexpression of miR-126 and miR-34a as tumor suppressive miRs increases anti-cancer efficacy in pancreatic adenocarcinoma (23). On the other hand, some miRNAs act as onco-miRs and upregulate in cancerous cells (24).  Upregulation of miR-21 as a onco-miR is correlated with chemotherapy resistance in a wide range of solid cancers such as pancreatic, prostate, ovarian, glioma, stomach and bladder cancers (25). Association between miR-21 and high proliferation, high invasion, low apoptosis, and metastatic potential has been indicated in cancer cell lines (26). The overexpression of miR-221/222 (27) and miR-155 (28, 29) the underlying mechanisms remain to be elucidated. In this study, we identified a high level of expression of miR-155 in a human lung adenocarcinoma A549R cell line that is highly resistant to ATO. We showed that the high level of miR-155 was associated with increased levels of cell survival, colony formation, cell migration and decreased cellular apoptosis, and this was mediated by high levels of Nrf2, NAD(P has been shown in different cancers such as breast, prostate, gastric and pancreatic cancers to be involved in cancer growth, migration, invasion, and inhibition of apoptosis. In addition, chemoresistance in cancer therapy has been found along with overexpression of miR-221/222 (30) and miR-155 (31).

Low solubility of silibilin in body is considered a problem in its use as a safe complementary herbal medicine for cancer. Herein, we encapsulated silibilin in polymersome NCs as SPNs and investigated the effects of SPNs on proliferation, migration and apoptosis in MIA PaCa-2 pancreatic cancer cell line. Our results demonstrated that SPNs induced apoptosis and inhibited cell growth and invasion through upregulation of several tumor suppressive miRs such as miR-34a, let-7b and miR-126a and downregulation of some onco-miRs such as miR-21, miR-221, miR-222 and miR-155. In addition, it seems that these miRNAs influence the anti-cancer effects of silibinin through upregulation of putative targets such as *P53*, *CASP*9, *APAF*1, and *BAX*, as well as downregulation of genes such as *BCL*-2, *CD34, AKT3, MASPINE, EGF, SERPINEA12, *and* BMP7.*

## Materials and Methods

***Materials***

Human pancreatic cancer cell lines MIA PaCa-2 (ATCC CRL­1420) were obtained from National Cell Bank of Iran (NCBI; Pasteur Institute of Iran). Materials used for cell culture such as DMEM medium and fetal bovine serum (FBS) were purchased from Gibico (Gaithersburg, MD). Silibinin was purchased from Sigma-Aldrich (Chemie GmbH, Germany). Oleyl choride and polyethylene glycil400 were purchased from Sigma-Aldrich (St. Louis, USA). Tri-ethylamine and chloroform were purchased from Millipore (Billerica, USA). The human monoclonal antibodies were purchased from thermos Fisher Afflymetrik Inc. (San Diego, CA, USA). 

***Cell culture***

Human pancreatic cancer cell lines MIA PaCa-2 were cultured in DMEM medium containing 10% FBS and 1% penicillin-streptomycin under standard culture conditions (37 ^°^C, in 95% humidified air containing 5% CO_2_).

***Generation of spheroids***

MIA PaCa-2 spheroids were formed by the hanging drop method (32, 33).To generate single-cell suspension, adherent cancer cells were detached with 0.05% trypsin-EDTA solution. 10 to 15 drops (each containing 20 µl) of harvested cells (0.5 ×10^6^ cells/ml) were placed on the lid of a 10 cm petri dish, which was then inverted over a dish (flipping the lid in gently) containing 5 ml of sterile phosphate-buffered saline (PBS) to protect the hanging drops from evaporation. For cell aggregation, the dish was incubated at 37 ^°^C for 48 hr, and drops were then gently transferred to a fresh plate coated by agar and filled with 10 ml media and incubated at 37 ^°^C for 24-48 hr. Spheroids aggregates were photographed by an inverted phase contrast microscope (Olympus, Japan). 

***Cancer stem cell (CSC) marker analysis***

For surface marker analysis by flow cytometry, first, the cells cultured in 6-well plate were dispatched by trypsin after 24 hrs. For cell surface labeling, the cells were incubated in 3% bovine serum albumin (BSA) (Sigma, St. Louis, US) for 10 min, were then fixed in 3% paraformaldehyde (Sigma, St. Louis, US) for 30 min, and were washed and incubated in 3% BSA for 10 min. The cells washed with 3% BSA were incubated with the human monoclonal CD133 antibody (thermoFisher Afflymetrik Inc., San Diego, CA, USA) for 30 min at 7 ^°^C. After washing the cell, a secondary human antibody (thermoFisher Afflymetrik Inc., San Diego, CA, USA) was added to cells. After two additional times of washing, primary conjugated antibodies against CD24, and CD44 were used and incubated for 30 min. Finally, the sample was subject to analysis using FACS Verse (BD Biosciences, San Jose, CA, USA). Side scatter and forward scatter profiles were used to eliminate cell doublets. At least 10,000 events were collected per sample, and the data were analyzed using WinMDI software (Mannheim, Germany). Positive cells were evaluated relative to the respective isotype control; Boolean gating was applied to determine the cells that co-expressed the CSC markers.

***Preparation of silibilin-encapsulated nanoparticles***

Oleoyl chloride (3.01 g, 0.01 mol) (Sigma-Aldrich, USA) and polyethylene glycol-400 (20 g, 0.01 mol) (Sigma-Aldrich, USA) were mixed and strificated to synthesize PEG_400_-OA in the presence of trimethylamine and chloroform as the solvent at 25 ^°^C for 4 hr. For purification of PEG_400_-OA, trimethylamine hydrochloride salt was filtered from organic phase and chloroform was evaporated in vacuum oven at 40 ^°^C for 4 hr. Then, silibilin was encapsulated in PEG_400_-OA carrier in a ratio of 1:6 by dissolving in acetone solution. After evaporation of acetone, sil/PEG_400_-OA (silibilin-encapsulated polymersome nanoparticles; SPNs) solution was filtered by syringe filter (220 nm) in dark condition.

***Cell proliferation and viability assay***

After physicochemical measurements, to confirm encapsulation of silibinin into polymersome (34) the viability of MIA PaCa-2 cells treated and untreated with SPNs was determined by MTT (3-(4, 5-dimethylthiazol-2-yl) 2, 5-diphenyl tetrazolium bromide) assay (35) (Sigma-Aldrich Co.) according to the manufacturer’s instructions. Briefly, 7×10^3^ cells/well were seeded in 96-well flat-bottomed tissue culture-untreated plates. After 24 hr, cultured cells were treated with different concentrations of SPNs (0, 5, 12.5, 25, 40, 45, 50, 70, 85, 100, 150 and 200 µg/ml). Then, MTT dye (0.5 mg/ml, [Sigma, St. Louis, USA]) was added to each well for 24, 48 and 72 hr and incubated at 37 ^°^C for 3 hr. To dissolve the formazan crystals, dimethyl sulfoxide (DMSO; 100 μl/well) was added and then, the optical density (OD) was measured at 570 nm using an ELISA plate reader (with a reference wavelength of 630 nm). Each experiment was performed for at least three times.

***Cell cycle assay***

For cell cycle analysis by flow cytometry based on propidium iodide (PI) staining protocol (36), MIA Paca-2 (0.5 × 10^6^) cells were seeded in 6-well plate and treated with different doses of SPNs (0, 30, 40, 50 µg/ml) for 24 hr. SPNs-treated and untreated cells were harvested, washed and re-suspended in PBS. The cells were fixed in ice-cold 70% ethanol and then stored at -20 ^°^C for ≥ 2 hr. The fixed cells were washed twice with PBS, suspended in 0.5 ml of cold PI (Sigma Aldrich) solution containing 10 µl RNase A (25 µg/ml, [Sinaclon Bio Science, Iran]), and 10 µl PI (50 µg/ml), and then incubated at 37 ^°^C for 30 min in the dark. Then, cell cycle analysis was performed using FACS calibur^TM^ flow cytometry system (BD Biosciences USA) and FlowJo7.6.1 software (Tree Star Inc., Ashland, USA).

***Apoptosis evaluation***

To analyze apoptosis induction, SPNs-treated and untreated cells (0, 30, 40, 50 µg/ml) were harvested after 24 hr and washed with PBS (0.01 M, pH 7.4) and re-suspended in binding buffer according to the manufacturer’s protocols (Annexin V- fluorescein isothiocyanate (FITC) kits (Miltenyi Biotec, Germany)). Annexin-V-FITC and PI mixture was added to cell pellet and incubated for 15 min at room temperature in dark condition. Then, percentage of apoptotic cells was determined by analyzing 15,000 UN gated cells using a FACS calibur^TM^ flow cytometry system (BD Biosciences san CA, USA) and FlowJo7.6.1 software. All experiments were performed in triplicate.

***DNA fragmentation assay***

DNA fragmentation assay was performed by agarose gel electrophoresis (37). In short, MIA PaCa-2 (10^6^ cells) were cultured under standard conditions. After 24 hr, cells were treated and untreated with 40 µg/ml of SPNs for 24 hr. A Total Fragment DNA Purification Kit (intron The MEGAquick-spin, South Korea) was used to extract DNA from SPNs-treated and untreated cells according to the manufacturer’s instructions. The extracted DNA (10 µg DNA samples) was electrophoresed on 2% agarose gels at 85 V for 90 min. Ethidium bromide was used to stain DNA. DNA fragmentation was evaluated by observation of sample in UV-transilluminator (Uvitec, UK)

***miRNA extraction and reverse transcription***

MIA Paca-2 (1×10^6^) cells were seeded in 25 ml flask and untreated and/or treated with 40 µg/ml of SPNs for 24 hr. Total RNA was extracted from cultured cells using Trizol reagent RNX-PLUS (Sinaclon Bio Science, Iran). Complementary DNA (cDNA) was synthesized using BONmiR™ qRT-PCR miRNA Detection Kit (Stem Cell Technology Research Center, Tehran, Iran) according to the manufacturer’s protocol. Quantitative real-time PCR was performed by SYBR Premix Ex Taq™ II (Takara bio, Japan) and monitored by Applied Biosystems^®^ StepOne^TM^ instrument and ABI7500 thermocycler according to this program: 95 ^°^C for 10 sec, 40 cycles at 95 ^°^C for 5 sec, 62 ^°^C for 20 sec, and finally, 72 ^°^C for 30 sec. The expression of miRNAs including miR-155, miR-222, miR-21, miR-34a, miR-126, let-7b and miR-221 were evaluated in SPNs-treated and untreated cells and normalized to SNORD47 gene as endogenous internal control. The used primers (Stem Cell Technology Research Center, Tehran, Iran) were listed in Table 1. All reactions were run at least in triplicate. The expression levels of miRNAs were analyzed using the equation 2^−ΔΔCT^.

***Prediction of potential targets of miRNAs***

The bioinformatics approach was used to identify the potential targets of miR-155, miR-222, miR-21, miR-34a, miR-126, let-7b and miR-221. Their target genes were predicted in apoptotic and migration pathways by some algorithms such as Target Scan (http://www.targetscan.org), miRWalk (http://zmf.umm.uniheidelberg.de/apps/zmf/mirwalk) and DIANA-microT (http://diana.imis.athena-innovation.gr.)

***Quantitative*** ***analysis*** ***of*** ***potential target*** ***genes***

The expression levels of potential target genes of miRNAs were measured by Q-RT-PCR. cDNA synthesis was carried out by BONmiR Detection kit (Stem Cell Technology Research Center, Tehran, Iran). Quantitative expression of target genes was analyzed using SYBR Premix ExTaq™II (Takara bio inc, Japan) in Applied Biosystems^®^ StepOne^TM^ instrument. The PCR program was performed as follows: 95 ˚C for 15 sec, 40 cycles at 95 ˚C for 5 sec and 60 ˚C for 30 sec. The used primers were listed in Table 1. The gene expression levels were normalized by beta2-microglobulin (B2M) gene as endogenous control. The fold change of genes was calculated by the 2^−ΔΔCT^ method. 

***Statistical analysis***

All experiments were repeated in at least three separate experiments, and results were measured as mean±standard deviations. The data analysis was performed by student’s t-test or one-way ANOVA followed by Tukey’s *post test*. *P*-value of 0.05 or less was considered to determine statistical significance.

## Results

***Stem cell characterization in MIA PaCa-2 cells***

**Figure 1:**
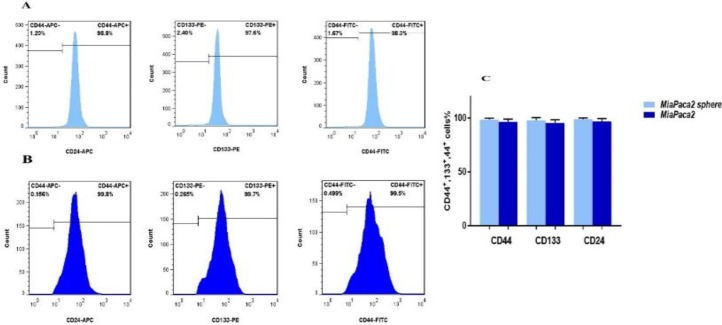
The expression of stem cell markers in MiaPaCa-2 sphere cells and the parental cells. Flowcytometric analysis of CD44, CD133 and CD24 in A) MIA PaCa-2 cells and B) MIA PaCa-2 sphere cells, C) Percentages of CD44, CD133 and CD24 positive cells in MIA PaCa-2 spheres compared to the parental cells (non-spheroid cells)

Spheroid formation capacity is the most widely known method to evaluate stemness in cancer cell cultures (38- the clinical need to overcome it, particularly for aggressive tumors such as pancreatic cancer, is very high. Aberrant activation of an epithelial-mesenchymal transition (EMT40). Hanging drop analysis demonstrated that MIA PaCa-2 cells had spheroid/colony formation capacity (Figure 1). CD44^+^, CD24^+^(41- 44) and CD133^+^ (45,44) are known as stem cell surface markers in pancreatic cancer cells. Flow cytometry analysis showed that 99.2% of MIA PaCa-2 cells expressed cell surface markers CD44 and CD133 (CD44^+^/CD133^+^). Moreover, 98.5% of MIA PaCa-2 cells were CD44^+^/CD24^+^***.***

***Effect of silibilin-encapsulated nanoparticles (SPNs) on viability*** ***of MIA PaCa-2 cells before and after hanging drop***

**Figure 2:**
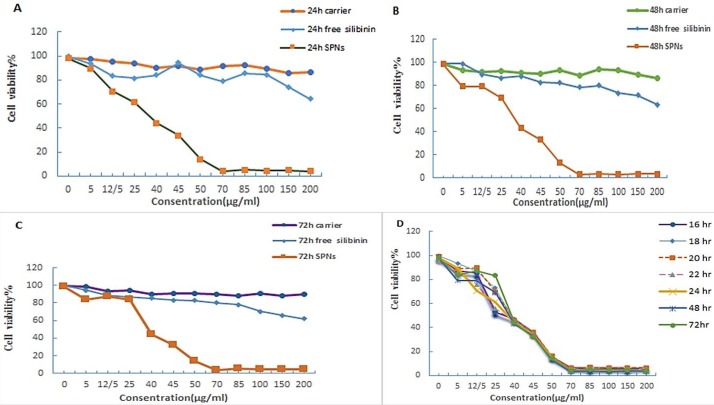
Cell viability of MIA PaCa-2 cells treated/untreated with SPNs. MIA PaCa-2 cells treated with empty nano-carrier (PEG_400_-OA), free silibinin and silibinin encapsulated in polymersome nanoparticles (SPNs) with different doses (0- 200 µg/ml) of each one for (A) 24 hr, (B) 48 hr, and (C) 72 hr, and MIA PaCa-2 cells treated with (D) different doses of SPNs (0- 200 µg/ml) were incubated for 16-72 hr. Viability of treated cells was measured by MTT assay. Results were representative of three experiments and each concentration was repeated at least three times in each experiment. The results are presented as mean±SD.* P-*value of 0.05 or less were considered significance

Our previous analysis showed that silibilin was encapsulated in polymersome nanoparticles (34). Appropriate ratio of silibilin to PEG_400_-OA based on drug loading, encapsulation efficiency and maximum drug dissolution without precipitation was obtained to be 1:6. Dynamic light scattering analysis of SPNs revealed an average diameter of 219.2 nm and an appropriate size distribution (PDI: 0.32). The zeta potential of SPNs was -12.15±1.20 mV. Encapsulation efficiency and drug loading content of 1 mg/ml SPNs were measured as 94.86±0.07 and 15.81±0.57, respectively (34). MIA PaCa-2 pancreatic cancer cells were cultured with different doses of SPNs (0-200 µg/ml). IC_50_ of SPNs was determined (40 µg/ml) by MTT assay after 24 hr. Our results showed that cell viability after treatment with SPNs decreased in a dose- and time-dependent manner. In addition, IC_50_ of SPNs on MIA PaCa-2 cells after 48 and 72 hr was determined to be 38 µg/ml and 36 µg/mlL, respectively. Our measurements demonstrated that cell viability decreased by less than 40% and 20%, respectively after treatment with 45 and 50 µg/ml of SPNs after 16 to 72 hr (Figure 2A)**. **Furthermore, our analysis showed that both free silibinin and SPN have cytotoxic effects on the MIA PaCa-2 cells, but free silibinin had lower cytotoxicity effects on treated cells rather than SPNs. Also, no significant cytotoxicity was demonstrated for empty NCs (even at 200 µg/ml) after 24, 48, and 72 hr of treatment (Figure 2A-C). 

**Figure 3:**
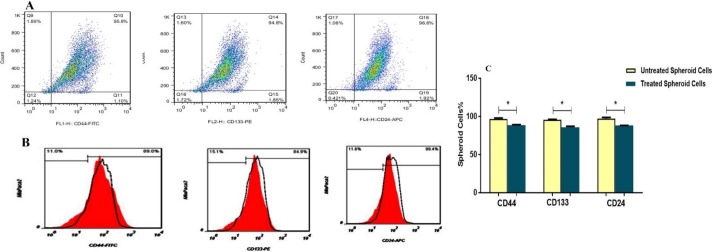
The expression pattern of Stem cell markers in MIA PaCa-2 Cells 48 hr after spheroid formation

**Figure 4:**
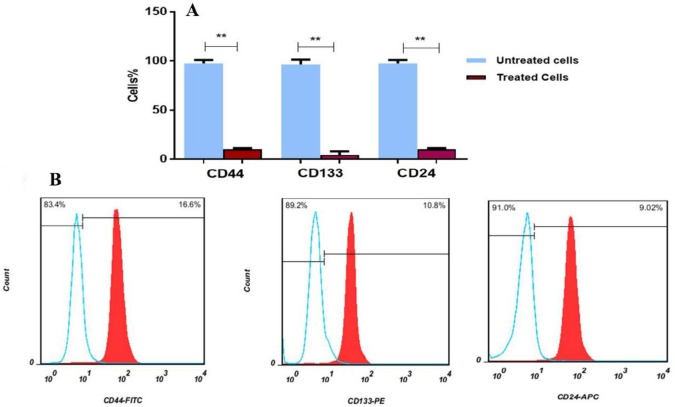
The expression pattern of stem cell markers in MIA PaCa-2 cells without hanging drop. A) Percentage of CD24+, CD44+ and CD133+ cells in silibinin encapsulated in polymersome nanoparticles (SPNs)-treated and untreated cells. B) Flow cytometry Histogram surface marker analysis of CD133, CD44 and CD24 markers in MIA PaCa-2 cells. Untreated (The red histogram) and treated with 40 µg/ml of SPNs after 24 hr (blue histogram). Asterisks indicate significant differences between the groups (*P<0.05)

**Figure 5:**
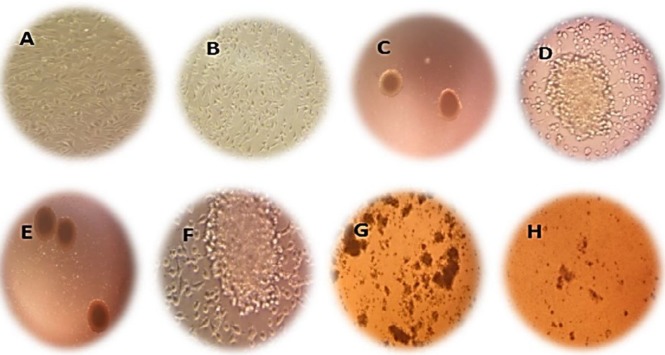
SPNs-treated MIA PaCa-2 cells before/after spheroid formation

FACS analysis showed that stem cell surface markers (CD44, CD133 and CD24) were decreased in spheroid MIA PaCa-2 cells treated with 40 µg/ml of SPNs (for 24 hr) compared to untreated cells (Figure 3). While, the expression levels of these markers were vigorously decreased in SPNs treated cells without hanging drop relative to SPNs untreated cells (Figure 4). Moreover, microscopy images showed that SPNs treatment for 24 hr destroyed spheroids MIA PaPa-2 cells (Figure 5).

***Cell-cycle analysis after treatment with SPNs in MIA PaCa-2 cancer cells***

**Figure 6.**
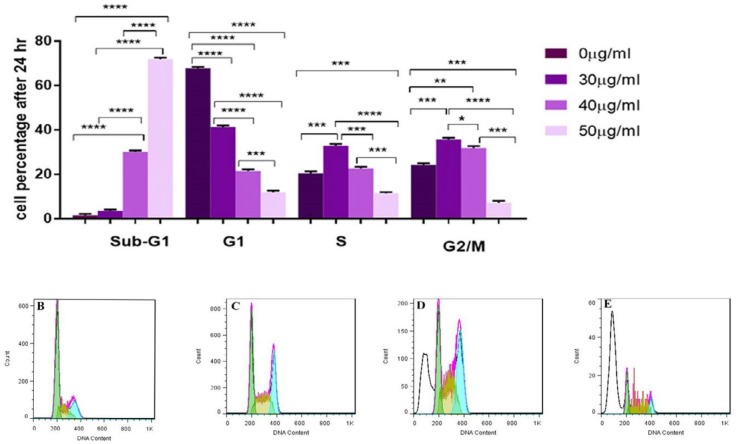
Cell cycle analysis. A) Percentage of cell phases after treatment with silibinin encapsulated in nanoparticles (SPNs) on different phases of cell cycle in MIA PaCa-2 cancer cells stained by propidium iodide (PI) and measured by flowcytometry. Photomicrographs showed percentage of apoptosis (sub-G1) and cell phases in control cells B), and the treated cells 30 µg/ml C), 40 µg/ml D) and 50 µg/ml E) of SPNs. The results are presented as mean±SD. Symbols indicate significant difference between cell groups (*P<0.05, **P<0.01, ***P<0.001 and ****P<0.0001)

Cell cycle distributions after treatment with 30, 40 and 50 µg/ml of SPNs after 24 hr were assessed by PI staining and flow cytometry analysis. Our findings showed that SPNs at doses of 30, 40 and 50 µg/ml induced apoptosis by 15.61%, 43.59% and 94.53%, respectively (Figure 6).

***Apoptosis Induction by SPNs***

**Figure 7:**
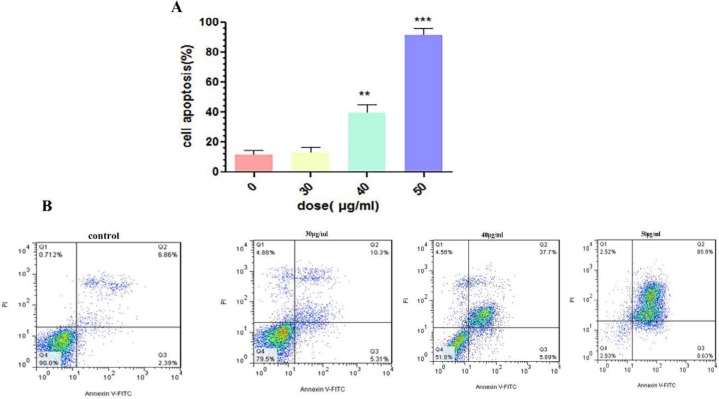
Flow cytometry analysis of SPNs-treated MIA PaCa-2 cells by Annexin V/PI double staining. Different doses of SPNs (30, 40, and 50 μg/ml) induced apoptosis in MIA PaCa-2 cells (A). Early (Annexin+, PI-) and late (Annexin+, PI+) apoptosis increased in B) untreated cells, and treated with C) 30 μg/ml, D) 40 μg/ml, and E) 50 μg/ml during 24 hr. Significance was determined using One way ANOVA variance followed by Tukey posttest (**P< 0.01, ***P < 0.001). SPNs: Silibinin encapsulated in polymersome nanoparticles

Treatment with IC_50_ dose of SPNs (40 µg/ml) and two close doses (30 and 50 µg/ml) was assessed to determine the percentage of apoptosis in treated and untreated cells. Our results showed that SPNs (40 µg/ml) induced apoptosis by ~40% in MIA PaCa-2 cancer cells. The percentage of early and late apoptosis after SPNs (40 µg/ml) induction were 37.7% and 5.89%, respectively. Apoptosis induction by 30 μg/ml of SPNs induced apoptosis in treated cells by less than 15%. Moreover, 50 µg/ml of SPNs in MIA PaCa-2 cancer cells increased apoptosis by more than 80% (Figure 7).

***DNA fragmentation after SPNs induction***

**Figure 8:**
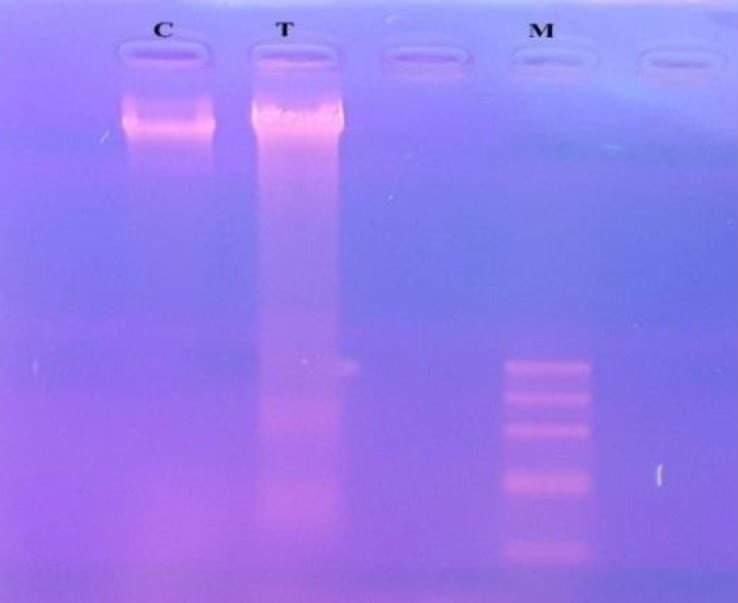
DNA fragmentation assay. MIA PaCa-2 cancer cells were treated and untreated with 40 µg/ml of SPNs for 24 hr

DNA fragmentation assay was used to detect the DNA damages in SPNs-treated and untreated MIA PaCa-2 cells. The effect of treatment with SPNs (40 µg/ml) on MIA PaCa-2 cells was observed in a DNA smear (Figure 8), indicating that DNA was damaged in treated cells in comparison with untreated ones.

***Deregulation of miRNAs in MIA PaCa-2 cells treated with SPNs***

**Figure 9:**
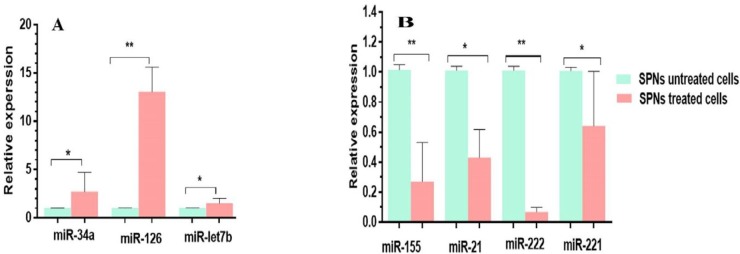
Relative expression of miRNAs in MIA PaCa-2 cells treated and untreated with 40 μg/ml of SPNs

Several miRNAs were evaluated in MIA PaCa-2 cancer cells after treatment with SPNs (40 µg/ml). Quantitative analysis by real-time PCR showed that the expression of miR-34a, miR-126 and miR-let7b increased between 1.49 to 8.64 folds in SPNs-treated cells compared to untreated ones. In addition, the expression of miR-155, miR-222 and miR-21 decreased between 0.068 to 0.42 folds (Figure 9).

***Deregulation of potential targets of miRNAs after SPNs induction***

**Figure 10:**
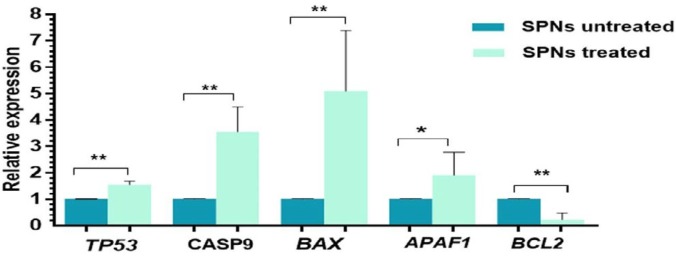
The expression pattern of potential targets of miRNAs in apoptotic pathway. Downregulation of several apoptotic genes and upregulation of an anti-apoptotic gene after SPNs (40 μg/ml) induction in MIA PaCa-2 cells. The results are represented as mean±SD. Symbols indicate significant difference between cell groups (*P<0.05, **P <0.001). SPNs: Silibinin encapsulated in polymersome nanoparticles

To have a better view about the abovementioned miRNAs in SPNs-treated cells, *in silico* analysis predicted their potential targets in apoptotic and cell proliferation and migration pathways (Table 2). The expression levels of five potential targets of the abovementioned miRNAs in apoptotic pathway were quantitatively analyzed in SPNs-treated and untreated cells. *CASP*-9, *p53*, *APAF1* and *Bax* levels were respectively upregulated in SPNs (40 μg/ml)-treated cells compared to untreated cells. The expression of *BCL2* significantly decreased with SPNs treatment (Figure 10).

***Downregulation of Genes Involved in Proliferation and Migration after SPNs Induction***

**Figure 11:**
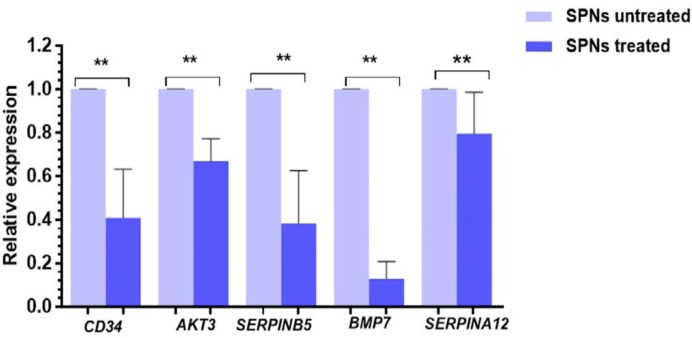
Quantitative expression of CD34, AKT3, MASPINE, EGF, SERPINEA12, and BMP7 in SPNs-treated and untreated MIA PaCa-2 cells (40 μg/ml) after 24 hr Relative expression of these genes was normalized to beta2-microglobulin (B2M). The re¬sults are represented as mean±SD. Symbols indicate a significant difference between cell groups (*P<0.05, **P<0.001). SPNs: Silibinin encapsulated in polymersome nanoparticles

The expression of several genes involved in migration in MIA PaCa-2 pancreatic cancer cells was quantitatively evaluated by real-time PCR. The pancreatic cancer cell line was treated with SPNs for 24 hrs. As shown in Figure 11, exposure to SPNs (40 μg/ml) led to downregulation of *CD34*, *AKT3*, *MASPINE*, *EGF*, *SERPINEA12*, and *BMP7* in MIA PaCa-2 cells***. ***

## Discussion

Nowadays, studies have focused on pharmacological effects of several herbal products such as silibilin in cancer therapy (46). In addition to killing cancer cells, some herbal drugs have the ability to overcome chemoresistance in treatment of cancer (44 -47). In addition, continuous consumption of medicinal herbs has been associated with the decrease of cancer risk as well as lack of cytotoxicity (46). As a herbal drug, silibilin is able to suppress bladder (49) and breast chemoresistance (44). Herein, we evaluated anti-cancer effect of SPNs on pancreatic MIA PaCa-2 cells. 

Many herbal compounds such as silibilin are insoluble in water and body fluids (50). To overcome this problem and better drug delivery to cells, researchers used different carriers such as nano-emulsions, liposomes, solid lipid nanoparticles (SLNs), micelles and lipid-dendrimer hybrid nanoparticles (51). The results of a study showed that silibilin-loaded poly(D, L-glycolide) (PLG)-PEG Fe_3_O_4 _nanoparticles had cytotoxicity effect on lung cancer compared to silibilin alone with dose- and time-dependent patterns (52). Yazdi Rouholamini *et al.* showed that silibinin-loaded niosomes and trimethyl-coated chitosan inhibited the growth of tumor cells and induced apoptosis more than silibinin alone in T47D cell line (53). In this study, we utilized polymersome nanoparticles for silibilin delivery to MIA PaCa-2 cells. PEG that is used in the formulation of drugs for PEGylate nanoparticles increases drug circulation half-time in body (51). In addition, OA as a natural vegetable oil has properties associated with non-toxicity, bio-compatibility, bio-degradability, permeability and bioavailability. Mono PEG conjugated with OA forms polymeric micelles and polymersomes that can be considered as a safe NCs for the delivery of small hydrophobic drugs (54). Due to having greater stability, storage capacity, release characteristics and plasma circulation times relative to their lipid counterparts (liposomes) (55), polymersomes can be suitable alternatives for drug delivery to cells. 

In a study, it has been revealed that silibinin without NCs had IC_50_ ranging from 200 to 570 ?M (~96 to 275 ?g/ml) in different breast cancer cell lines (47).?Maleki Zadeh μM (~96 to 275 μg/ml) in different breast cancer cell lines (47). Maleki Zadeh *et al.* showed that silibinin can inhibit the growth of MCF-7 (1) and T47-D cells (56) and induce apoptosis by IC_50_~100 µg/ml after 24 hr. A significant decrease in cell proliferation in Panc-1 and Bxpc-1 pancreatic cancer cells was observed after treatment with 200 μM (~96 μg/ml) of silibinin for 48 hr (7). IC_50_ of silibinin in AsPC-1 pancreatic cancer cells was determined to be 224.20 and 87.25 µM after 48 and 72 hr, respectively (57) cyclin E2, cyclin A and cyclin B1 were decreased. The expression of G1-associated cell cycle-dependent kinases, cyclin-dependent kinase (CDK. Also, Hossainzadeh *et al.* reported that SPNs (~45 μg/ml) inhibited cell proliferation in MDA-MB-231 breast cancer cells after 24 hr (34). In our study, 40 μg/ml of silibinin in nanoparticle structure was able to inhibit the proliferation of MIA PaCa-2 cells after 24 hrs. Thus, using polymersome nanoparticles increased delivery of silibinin to cancer cells with lower concentration at a shorter period.

The use of nanotechnology in the pharmaceutical industry greatly expands the scope of the existing anti-cancer drugs and strategies for treatment, especially in the field of targeting CSCs (58). Zhou *et al.* showed that chemo-sensitivity of CSCs, isolated from MBA-MB-231 and MCF-7 cell lines, was improved by treatment with curcumin as a herbal compound (59). In a study, Atashpour *et al.* determined that quercetin, as a flavonoid secondary metabolite, inhibited the proliferation of CD133^+^ CSCs harvested from HT29 cell lines and induced apoptosis, and enhanced the sensitivity of these cells to doxorubicin (60). In our study, stem cell markers (CD44, CD24 and CD133) decreased after SPNs induction in MIA PaCa-2 cancer cells. It is considerable that in spheroid MIA PaCa-2 cells, since SPNs can only be uptaken by surface cells and not by deep part of cells, the percentage of stem cell markers decreased less than non-spheroid ones. Concurrent expression of CD44 and CD24 in the pancreatic CSCs is very tumorigenic and helps them renew themselves and create a distinct generation (41). The expression of CD133 marker in the surface of pancreatic cancer cell lines increases proliferative capacity as a property of the CSCs (61),(60). Almanaa *et al.* suggested that curcumin, as a herbal drug, leads to their depletion through induction of differentiation in CSCs (62). Herein, SPNs decrease CSCs markers on the surface of treated cells, and it seems that this effect is an anti-tumorogenesis property of silibinin. 

Silibinin induced a strong dose-dependent G1 arrest in BxPC-3 pancreatic cancer cells and a moderate response in advanced PANC-1 pancreatic cancer cells (63). In a study, silibinin decreased cell percentage in S phase and increased cell cycle arrest in G1 phase in AsPC-1 cells, but not in BxPC-3 and Panc-1 pancreatic cancer cells (7). Our analysis indicated that SPNs led to the decrease in cell percent in the G1, S, and G2/M phases as well as the increase in cells that entered sub-G1 phase. It seems that doses of SPNs up to 30 μg/ml may induce S and G2/M arrest, but at doses ≥40 μg/ml strongly induce cell death; the cell cycle arrest was not observed at these doses. 

Ge *et al.* showed that 200 μM of silibinin induced apoptosis in AsPC-1, Panc-1 and BxPC-3 pancreatic cancer cells less than 40% after 48 hr (7). In addition, apoptotic rate of SW1990 pancreatic cancer cells treated with 200 µM of silibinin was 27.69% after 48 hrs. In this study, apoptosis analysis confirmed SPNs-induced programmed cell death in MIA PaCa-2 cancer cells in lower concentration of silibinin compared to previous studies. Our analysis suggested that applying ≥50 μg/ml doses of SPNs may have vigorous cytotoxic effect on pancreatic cancerous cells and CSCs. 

Nowadays, miRNAs are known as important regulators in signaling pathways governing stem-cell fate. Therefore, external cell stimuli like different drugs may affect cells partly through deregulation of their miRNAs. Herbal drugs with anti-cancer effects can upregulate tumor suppressive miR and downregulate onco-miR. MiR-155 knock-down leads to suppression of cell growth and colony formation as well as downregulation of epidermal growth factor receptor (EGFR), membrane-type 1 matrix metalloproteinase (MT1-MMP), and Kirsten rat sarcoma viral oncogene homolog (K-Ras) in pancreatic cancer (64). Overexpression of miR-21 in pancreatic cancer cells is positively associated with the overexpression of invasion-related genes, including MMP-2, MMP-9 and vascular endothelial growth factor (VEGF) (65). Overexpression of miR-221/miR-222 was observed in different grades of pancreatic intraepithelial neoplasia (PanIN) lesion and suggested its roles in progression of pancreatic cancer (66). Herein, underexpression of onco-miRs, including miR-155, miR-21, miR-221 and miR-222 after SPNs treatment showed that silibinin partly functioned through suppression of the abovementioned onco-miRs on the inhibition of proliferation, and induction of apoptosis in MIA PaCa-2 cells. 

On the other side, underexpression of some tumor suppressive miRs such as miR-34a, let7b and miR-126 has been indicated in pancreatic cancer cells (67). Ectopic expression of miR-34a, as tumor suppressive miR, caused downregulation of Bcl-2, Notch1, and Notch2 and also inhibition of cell proliferation and invasion, induction of apoptosis and cell cycle arrest in pancreatic cancer cells (66). Downregulation of let-7 in pancreatic cancer is associated with increase in chemoresistance (68). It seems that silibinin might overcome chemoresistance in cancer therapy partly through overexpression of let-7b. Tumor suppressive miR-126 and its target ADAM9 play role in controlling migration and invasion in pancreatic cancer. MiR-126 is also the target of other important oncogenes such as KRAS and CRK in pancreatic cancer (69) Therefore, overexpression of miR-126 in SPNs-treated cells can be a reason for inhibition of MIA PaCa-2 proliferation and migration. The transfection of MIA PaCa-2 cells with miR-34 led to reduction in CD44^+^/CD133^+^ cells and reduction in spheroid formation (70). The upregulation of Notch-1 in pancreatic cancer cells induces overexpression of miR-21 and underexpression of let-7a and let-7b (71). Thus, in this study, silibinin was able to suppress stem cell markers on MIA PaCa-2 cells and inhibit spheroid formation and CSCs progression through downregulation of some miRNAs such as miR-21 and upregulation of other miRNAs such as miR-34a and let-7b. 

Nowadays, to have a better insight into miRNAs function in tumorigenesis, computational approaches predict putative targets of miRNAs in different biological pathways. Our *in silico* analysis predicted putative targets of miR-21, miR-221, miR-222 and miR-155 in apoptotic pathways. Overexpression of onco-miR miR-21 was correlated with downregulation of pro-apoptotic Bax and upregulation of anti-apoptotic Bcl2 and induced apoptosis in glioblastoma (72). As a pro-apoptotic protein, p53 upregulated modulator of apoptosis (PUMA) is an important mediator of p53-associated apoptosis. Mover, PUMA indirectly activated Bax through binding and inactivating Bcl2. The knockdown of miR-221/222 decreases Bcl2 and increases Bax in glioblastoma cells. Thus, miR-221/222 are negative regulators of PUMA that lead to downregulation of Bcl2 and upregulation of BAX (73) In addition, apoptotic protease activating factor-1 (APAF-1) is a validated target of miR-155(74). APAF-1, cytochrome c and caspase 9, apoptosis-related cysteine peptidase (CASP-9) are members of apoptosome and play role in intrinsic pathway of apoptosis (1). In addition, miR-155 leads to apoptosis inhibition through inactivation of Bax and caspase-9 and activation of Bcl-2 (75). It appears that SPNs lead to upregulation of pro-apoptotic genes such as caspase -9, P53, APAF1 and Bax directly or indirectly through downregulation of onco-miRAs such as miR-21, miR-221, miR-222 and miR-155. On the other hand, miR-155 confers radioresistance to cancerous cells (74).

In addition, overexpression of Bcl-2 in cancer cells causes resistance to apoptosis induction and results in chemoresistance (72)their function mainly represses the target mRNAs transcripts via imperfectly complementary to the 3’UTR of target mRNAs. Several miRNAs have been recently reported to be involved in modulation of glioma development, especially some up-regulated miRNAs, such as microRNA-21 (miR-21. Thus, it seems that as a complementary anti-cancer drug, silibinin can overcome chemoresistance in cancer therapy through downregulation of miRNAs such miR-155 and Bcl-2. 

Maspin is a member of the Serpin family (Serine protease inhibitor) (76)that plays role in apoptosis, and angiogenesis in breast, lung and prostate cancers through preventing cell motility, invasion, and metastasis (76, 77). Akt (Protein kinase B, PKB) is a serine/threonine kinase that plays a key role in regulating cell survival, insulin signaling, angiogenesis and tumor formation. Downregulated Akt3 isoform prevents *in vitro* ovarian cancer cell proliferation, colony formation and migration (78). Recent studies have shown that Akt3 is responsible for embryonic stem cells (ESC) survival and G1/S-transition mechanism by suppression of p53 activity (79). Serpin Family A Member 12 (SERPINA12), also known as Vaspin, may be involved in carcinogenicity and its down-expression that leads to increased apoptosis in treated cells. BMPs act as tumor suppressors and oncogene-induced tumorigenesis factor. BMP7 in breast cancer acts as tumor suppressor and stimulates VEGF expression in prostate cancer (80). CD34 may play a role in the attachment of stem cells to the bone marrow extracellular matrix or to stromal cells (81). Defects in adhesion or migration may be the effects of CD34 expression on cell differentiation and proliferation (82). These genes were potential targets of miRNAs 34a, let7b and 126 that were downregulated in SPNs-treated cells. Our finding suggested that SPNs can function as an anti-tumorogenesis agent through different mechanisms.

**Table1:** Primers used for Q-RT-PCR

**Name**	**Sequence**
**hsa-mir-21-5p F**	**5'-GGCTTGTCAGACTGATGTTG**
**hsa-miR-221-3p-F**	**5’-ATTCAGGGCTACATTGTCTG**
**hsa-miR-222-3p –F**	**5’-ACGATGCCAGTTGAAGAAC**
**hsa-miR-155-5p**	**5’-ACTTGGCTAATCGTGATAGG**
**hsa-let-7b-F**	**5’-GCGTGAGGTAGTAGGTTGTG**
**hsa-miR-34a-F**	**5’-ATGGTGGCAGTGTCTTAGC**
**hsa-miR-126-3PF**	**5’-CAGCGTACCGTGAGTAATG**
**Common Reverse primer**	**5'-GAGCAGGGTCCGAGGT**
***Beta2M - F***	**5'-** ***ATG CCT GCC GTG TGA AC***
***Beta2M - R***	**5'-** ***ATC TTC AAA CCT CCA TGA TG***
***P53 - F***	**5'-** ***GGA GTA TTT GGA TGA CAG AAA C***
***P53 - R***	**5'-** ***GAT TAC CAC TGG AGT CTT C***
***BCL2-F***	**5'-** ***GATAACGGAGGCTGGGATG***
***BCL2-R***	**5'-** ***CAGGAGAAATCAAACAGAGGC***
***EGF- F***	**5'-** ***TTT TGT TGT TCC TGC AGC CC***
***EGF- R***	**5'-** ***GCA AAA TCA TCA GCA TGG ACC***
***BAX-F***	**5'-** ***CAA ACT GGT GCT CAA GGC***
***BAX-R***	**5'-** ***CAC AAA GAT GGT CAC GGT C***
***AKT3-F***	**5'-** ***TCTCTGCCTTGGACTATCTAC***
***AKT3-R***	**5'-** ***TCATTATCTTCTAACACCTCTGG***
***maspin -F***	**5'-** ***TGT GGT TAA TGC TGC CTA C***
***maspin -R***	**5'-** ***GTT TGG TGT CTG TCT TGT TG***
***SERPINA12-F***	**5'-** ***GCT GGG TTC CTC TCT TTT C***
***SERPINA12-R***	**5'-** ***TTG AAG AAT ATC CTC ATT CCT AG***
***BMP7-F***	**5'-** ***CAG ACG CTG GTC CAC TTC***
***BMP7-R***	**5'-** ***CGG AGA TGG CAT TGA GC***
***CD34-F***	**5'-** ***ACC CCA GAG TTA CCT ACC CAG***
***CD34-R***	**5'-** ***TGT CGT TTC TGT GAT GTT TGT TG***

Flow cytometry Dot-Plot and Histogram surface marker analysis of CD133, CD44 and CD24 markers in MIA PaCa-2 spheroid cells after 48 hr. A) Untreated (The black histogram) and B) treated with 40 µg/ml of silibinin encapsulated in polymersome nanoparticles (SPNs) after 24 hr (The red histogram). C) Percentage of CD24+, CD44+ and CD133+ cells in spheroid cells treated and untreated with SPNs. Asterisks indicate significant differences between the groups (*P<0.05)

MIA PaCa-2 cells without hanging drop as control with magnification A) 200x, and B) 400x. Spheroids formation of MIA PaCa-2 cells after 48 hr at hanging drops condition C) 200x, and D) 400x magnification. Spheroids formation of MIA PaCa-2 cells after 72 hr at hanging drops condition E) 200x and F) 400x magnification. Treatment of spheroids with SPNs (40 µg/ml) during G) 48 hr (200x magnification), and H) 72 hr (200x magnification). SPNs: Silibinin encapsulated in polymersome nanoparticles

DNA fragmentation in cells treated with (T) 40 µg/ml SPNs and (C) untreated ones was determined with DNA gel electrophoresis. M: Molecular weight marker (100 bp), SPNs: Silibinin encapsulated in polymersome nanoparticles

Relative expression of miRNAs was normalized to SNORD47 as an endogenous control. Representative data from at least three triplicate experiments are shown. The re¬sults are represented as mean±SD. Symbols indicate a significant difference between cell groups (*P<0.05, **P <0.001). SPNs: Silibinin encapsulated in polymersome nanoparticles

**Table 2:** Some potential targets of miRNAs in apoptotic and migration pathways

***microRNA***	***Potential target***	***Gene name***
***21***	***APAF1***	***Apoptotic peptidase activating factor 1***
	***ARHGAP24***	***Rho GTPase activating protein 24***
	***EGR3***	***early growth response 3***
	***MAP2K3***	***mitogen-activated protein kinase kinase 3***
	***MEGF9***	***multiple EGF-like-domains 9***
	***MYCL***	***v-myc avian myelocytomatosis viral oncogene lung carcinoma derived ho*** ***mo*** ***log***
	***PIK3R1***	***phosphoinositide-3-kinase, regulatory subunit 1 (alpha)***
	***RASA1***	***RAS p21 protein activator (GTPase activating protein) 1***
	***RGS7BP***	***regulator of G-protein signaling 7 binding protein***
	***SMAD7***	***SMAD family member 7***
	***TADA2A***	***transcriptional adaptor 2A***
	***TGFBI***	***transforming growth factor, beta-induced, 68kDa***
	***TP53***	***tu*** ***mo*** ***r protein p53***
***221***	***APAF1***	***Apoptotic peptidase activating factor 1***
	***BAG1***	***BCL2-associated athanogene ***
	***CADM1***	***cell adhesion *** ***mo*** ***lecule 1 ***
	***CASP2***	***caspase 2, apoptosis-related cysteine peptidase ***
	***CASP3***	***caspase 3, apoptosis-related cysteine peptidase ***
	***CASP9***	***caspase 9, apoptosis-related cysteine peptidase ***
	***CCNG1***	***cyclin G1 ***
	***CRTC1***	***CREB regulated transcription coactivator 1 ***
	***DAP***	***death-associated protein ***
	***GPRIN2***	***G protein regulated inducer of neurite outgrowth 2 ***
	***GRAP***	***GRB2-related adaptor protein ***
	***GRB2***	***growth factor receptor-bound protein 2 ***
***.***	***JAK2***	***Janus kinase 2 ***
	***NOTCH1***	***notch 1 ***
	***TAF13***	***TAF13 RNA polymerase II, TATA box binding protein (TBP)-associated factor, 18kDa ***
	***TP53AIP1***	***tu*** ***mo*** ***r protein p53 regulated apoptosis inducing protein 1 ***
***222***	***APAF1***	***Apoptotic peptidase activating factor 1***
	***CADM1***	***cell adhesion *** ***mo*** ***lecule 1 ***
	***CASP3***	***caspase 3, apoptosis-related cysteine peptidase ***
	***CASP9***	***caspase 9, apoptosis-related cysteine peptidase ***
	***CCNG2***	***cyclin G2 ***
	***CCPG1***	***cell cycle progression 1 ***
	***CD34***	***CD34 *** ***mo*** ***lecule ***
	***CDK1***	***cyclin-dependent kinase 1 ***
	***CREBBP***	***CREB binding protein ***
	***DFFA***	***DNA fragmentation factor, 45kDa, alpha polypeptide ***
	***FGFR1OP***	***FGFR1 oncogene partner ***
	***GAB3***	***GRB2-associated binding protein 3 ***
	***GRK5***	***G protein-coupled receptor kinase 5 ***
	***MAD2L1***	***MAD2 mitotic arrest deficient-like 1 (yeast) ***
	***MEGF11***	***multiple EGF-like-domains 11 ***
	***NOTCH2***	***notch 2 ***
	***NOX1***	***NADPH oxidase 1 ***
	***RASAL2***	***RAS protein activator like 2 ***
	***SERBP1***	***SERPINE1 mRNA binding protein 1 ***
	***SMAD2***	***SMAD family member 2 ***
	***TANK***	***TRAF family member-associated NFKB activator ***
	***TP53INP1***	***tu*** ***mo*** ***r protein p53 inducible nuclear protein 1 ***
***155***	***BAG5***	***BCL2-associated athanogene 5 ***
	***DMTF1***	***cyclin D binding myb-like transcription factor 1 ***
	***E2F2***	***E2F transcription factor 2 ***
	***FGF9***	***fibroblast growth factor 9 ***
	***G3BP2***	***GTPase activating protein (SH3 domain) binding protein 2 ***
	***GAB3***	***GRB2-associated binding protein 3 ***
	***GDF6***	***growth differentiation factor 6 ***
	***KRAS***	***Kirsten rat sarcoma viral oncogene ho*** ***mo*** ***log ***
	***KSR1***	***kinase suppressor of ras 1 ***
	***MARK2***	***MAP/microtubule affinity-regulating kinase 2 ***
	***PKIA***	***protein kinase (cAMP-dependent, catalytic) inhibitor alpha ***
	***RBAK***	***RB-associated KRAB zinc finger ***
	***SLA***	***Src-like-adaptor ***
	***SMAD2***	***SMAD family member 2 ***
	***SOCS1***	***suppressor of cytokine signaling 1 ***
	***TAB2***	***TGF-beta activated kinase 1/MAP3K7 binding protein 2 ***
	***TAPT1***	***transmembrane anterior posterior transformation 1 ***
	***TBRG1***	***transforming growth factor beta regulator 1 ***
	***TCF4***	***transcription factor 4 ***
	***TCF7L2***	***transcription factor 7-like 2 (T-cell specific, HMG-box) ***
	***TP53INP1***	***tu*** ***mo*** ***r protein p53 inducible nuclear protein 1 ***
	***TPD52***	***tu*** ***mo*** ***r protein D52 ***
	***TRAF3***	***TNF receptor-associated factor 3 ***
	***VEZF1***	***vascular endothelial zinc finger 1 ***
***126***	***AKT3***	***v-akt murine thy*** ***mo*** ***ma viral oncogene ho*** ***mo*** ***log 3 ***
	***ALDH1A1***	***aldehyde dehydrogenase 1 family, member A1 ***
	***ANGPT1***	***angiopoietin 1 ***
	***BAK1***	***BCL2-antagonist/killer 1 ***
	***BCL2***	***B-cell CLL/lymphoma 2 ***
	***CADM1***	***cell adhesion *** ***mo*** ***lecule 1 ***
	***CCNG1***	***cyclin G1 ***
	***CCNG2***	***cyclin G2 ***
	***CCPG1***	***cell cycle progression 1 ***
	***CREBBP***	***CREB binding protein ***
	***DFFA***	***DNA fragmentation factor, 45kDa, alpha polypeptide ***
	***MYCBP***	***MYC binding protein ***
	***MYCT1***	***myc target 1 ***
	***NKAP***	***NFKB activating protein ***
	***RASA1***	***RAS p21 protein activator (GTPase activating protein) 1 ***
	***RASAL2***	***RAS protein activator like 2 ***
	***RASEF***	***RAS and EF-hand domain containing ***
	***RASGEF1B***	***RasGEF domain family, member 1B ***
	***RGS1***	***regulator of G-protein signaling 1 ***
	***SERPINA10***	***serpin peptidase inhibitor, clade A (alpha-1 antiproteinase, antitrypsin), member 10 ***
	***SERPINA5***	***serpin peptidase inhibitor, clade A (alpha-1 antiproteinase, antitrypsin), member 5 ***
	***SMAD2***	***SMAD family member 2 ***
	***TAB2***	***TGF-beta activated kinase 1/MAP3K7 binding protein 2 ***
	***TAPBP***	***TAP binding protein (tapasin) ***
	***TNIK***	***TRAF2 and NCK interacting kinase ***
	***VEGFA***	***vascular endothelial growth factor A ***
***34a***	***AKT3***	***adenylate kinase 3***
	***SERPINB5***	***Maspin (mammary serine protease inhibitor)***
	***BMP7***	***bone *** ***mo*** ***rphogenetic protein 7***
	***CD34***	***CD34 *** ***mo*** ***lecule***
	***EGF***	***Epidermal growth factor***
	***ARHGAP26***	***Rho GTPase activating protein 26 ***
	***ARPP19***	***cAMP-regulated phosphoprotein, 19kDa ***
	***BCL2***	***B-cell CLL/lymphoma 2 ***
	***CDC25A***	***cell division cycle 25A ***
	***E2F3***	***E2F transcription factor 3 ***
	***FOSL1***	***FOS-like antigen 1 ***
	***GPR12***	***G protein-coupled receptor 12 ***
	***GREM2***	***gremlin 2, DAN family BMP antagonist ***
	***MDM4***	***Mdm4 p53 binding protein ho*** ***mo*** ***log (*** ***mo*** ***use) ***
	***NOTCH1***	***notch 1 ***
	***SMAD4***	***SMAD family member 4 ***
	***SOCS4***	***suppressor of cytokine signaling 4 ***
***Let7b***	***AKAP5***	***A kinase (PRKA) anchor protein 5 ***
	***AMER3***	***APC membrane recruitment protein 3 ***
	***ANGPTL2***	***angiopoietin-like 2 ***
	***CDCA8***	***cell division cycle associated 8 ***
	***E2F2***	***E2F transcription factor 2 ***
	***HBEGF***	***heparin-binding EGF-like growth factor ***
	***MAP3K1***	***mitogen-activated protein kinase kinase kinase 1, E3 ubiquitin protein ligase ***
	***MDM4***	***Mdm4 p53 binding protein ho*** ***mo*** ***log (*** ***mo*** ***use) ***
	***RASGRP1***	***RAS guanyl releasing protein 1 (calcium and DAG-regulated) ***
	***RASL10A***	***RAS-like, family 10, member A ***
	***RGS16***	***regulator of G-protein signaling 16 ***
	***SMAD2***	***SMAD family member 2 ***
	***SOCS1***	***suppressor of cytokine signaling 1 ***
	***STK24***	***serine/thrseonine kinase 24 ***
	***SERPINA12***	***Serpin Family A Member 12***
	***SERPINB5***	***Maspin (mammary serine protease inhibitor)***
	***BMP7***	***bone *** ***mo*** ***rphogenetic protein 7***
	***CD34***	***CD34 *** ***mo*** ***lecule***
	***EGF***	***Epidermal growth factor***

## Conclusion

Polymersome nanoparticles were used for the delivery of silibinin in MIA PaCa-2 cells. Our quantitative analysis showed downregulation of some miRNAs such as miR-21 and upregulation of miRNAs such as miR-34a and let-7b in SPNs-treated cells. Moreover, the decrease in stem cell markers on MIA PaCa-2 cells and inhibition of spheroid formation suggested that SPNs may inhibit CSCs progression. On the other hand, upregulation of some apoptotic genes and downregulation of genes involved in migration of potential targets of up/downregulated miRNAs in SPNs-treated cells confirmed the important role of silibinin in apoptosis induction and migration inhibition. 
